# The development of a machine learning model to train junior ophthalmologists in diagnosing the pre-clinical keratoconus

**DOI:** 10.3389/fmed.2024.1458356

**Published:** 2024-09-18

**Authors:** Yang Jiang, Hanyu Jiang, Jing Zhang, Tao Chen, Ying Li, Yuehua Zhou, Youxin Chen, Fusheng Li

**Affiliations:** ^1^Department of Ophthalmology, Peking Union Medical College Hospital, Chinese Academy of Medical Sciences, Beijing, China; ^2^Key Laboratory of Ocular Fundus Diseases, Peking Union Medical College, Chinese Academy of Medical Sciences, Beijing, China; ^3^Eight-Year Medical Doctor Program, Peking Union Medical College, Chinese Academy of Medical Sciences, Beijing, China; ^4^Department of Ophthalmology, Beijing Ming Vision Clinic, Beijing, China; ^5^Department of Ophthalmology, Beijing Fenglian Jiayue Lege Clinic, Beijing, China

**Keywords:** machine learning, preclinical keratoconus, training, education, keratoconus

## Abstract

**Purpose:**

This study aims to evaluate the diagnostic performance of a machine learning model (ML model) to train junior ophthalmologists in detecting preclinical keratoconus (PKC).

**Methods:**

A total of 1,334 corneal topography images (The Pentacam HR system) from 413 keratoconus eyes, 32 PKC eyes and 222 normal eyes were collected. Five junior ophthalmologists were trained and annotated the images with or without the suggestions proposed by the ML model. The diagnostic performance of PKC was evaluated among three groups: junior ophthalmologist group (control group), ML model group and ML model-training junior ophthalmologist group (test group).

**Results:**

The accuracy of the ML model between the eyes of patients with KC and NEs in all three clinics (99% accuracy, area under the receiver operating characteristic (ROC) curve AUC of 1.00, 99% sensitivity, 99% specificity) was higher than that for Belin-Ambrósio enhanced ectasia display total deviation (BAD-D) (86% accuracy, AUC of 0.97, 97% sensitivity, 69% specificity). The accuracy of the ML model between eyes with PKC and NEs in all three clinics (98% accuracy, AUC of 0.96, 98% sensitivity, 98% specificity) was higher than that of BAD-D (69% accuracy, AUC of 0.73, 67% sensitivity, 69% specificity). The diagnostic accuracy of PKC was 47.5% (95%CI, 0.5–71.6%), 100% (95%CI, 100–100%) and 94.4% (95%CI, 14.7–94.7%) in the control group, ML model group and test group. With the assistance of the proposed ML model, the diagnostic accuracy of junior ophthalmologists improved with statistical significance (*p* < 0.05). According to the questionnaire of all the junior ophthalmologists, the average score was 4 (total 5) regarding to the comprehensiveness that the AI model has been in their keratoconus diagnosis learning; the average score was 4.4 (total 5) regarding to the convenience that the AI model has been in their keratoconus diagnosis learning.

**Conclusion:**

The proposed ML model provided a novel approach for the detection of PKC with high diagnostic accuracy and assisted to improve the performance of junior ophthalmologists, resulting especially in reducing the risk of missed diagnoses.

## Introduction

Keratoconus (KC) is a noninflammatory condition. This resulted in irregular astigmatism and reduced vision (1). A recent epidemiological study demonstrated that KC is not a rare disease, with a prevalence of 1.2% (2). However, early detection of KC is challenging. In the earliest stages, preclinical keratoconus (PKC) does not show classical keratometric or biomicroscopic features. Misdiagnosis of PKC leads to an increased risk of iatrogenic ectasia after refractive surgery (3), which is the most severe and irreversible complication after corneal refractive surgery (4). It has been estimated that 50% of patients with PKC progress to KC within 16 years (5). In addition, with the availability of corneal cross-linking, early detection can also contribute to the delay or cessation of KC progression (6).

However, the detection of early stage of keratoconus is a challenging task, especially for the junior ophthalmologists. We develop a machine learning system using corneal topography images obtained from three clinics to detect PKC and use it to train the junior ophthalmologists, helping them improve their accuracy of PKC detection.

## Methods

### Study design and population

The study followed the tenets of the Declaration of Helsinki. The study protocol was approved by the Review Board and Human Ethics Committee of Peking Union Medical College Hospital, Chinese Academy of Medical Sciences. The Pentacam HR system (Oculus, Wetzlar, Germany) is a Scheimpflug priciple-based tomography system with a rotating camera that can record 25,000 corneal points. Five images obtained by the Pentacam HR system were analyzed using for analyses by an artificial intelligence (AI) model: anterior and posterior corneal curvatures, anterior and posterior elevations, and entire pachymetry distribution.

Both eyes of all patients were evaluated using the Pentacam HR system. Patients were classified into KC, PKC, or NE groups. KC eyes were diagnosed using the following criteria established by the Collaborative Longitudinal Evaluation of Keratoconus (CLEK) study (7): (1) at least one of the following slit-lamp signs: focal stromal thinning, Vogt’s strias, Fleischer’s ring >2-mm arc, or corneal scarring consistent with KC; (2) irregular cornea with focal steeping determined via distorted keratometry test, and distortion of the retinoscopic or ophthalmoscopic red reflex and (3) no history of contact lens wear, ocular surgery or extensive scarring.

According to the Global consensus on keratoconus and ectatic diseases (8), true unilateral keratoconus does not exist. Therefore, PKC eyes were the unaffected eyes of the keratoconus patients whose the other eye were diagnosed with KC. An explicit definition of subclinical keratoconus (SKC) is lacking. The most common definition of SKC used to be patients with a tomographic pattern of localized steepening in the posterior or anterior corneal surface or paracentral corneal thinning, but no clinical signs of KC. However, PKC refers to unaffected eye of keratoconus patient with no sign of keratoconus by tomography. PKC eyes were diagnosed using the following criteria established by the Keratoconus Severity Score (KSS) study (9): (1) No corneal scarring consistent with KC; (2) No slit-lamp signs for keratoconus; (3) Inferior or superior steepening no more than 3D steeper than average corneal power (ACP); (4) ACP no more than 48D.

The eyes of normal individuals undergoing routine ophthalmological examination met the following screening criteria (10): (1) normal clinical evaluations (defined as no clinical signs or suggestive topographic patterns for suspicious KC, KC, or pellucid marginal degeneration), (2) no family history of KC, (3) no history of ocular surgery or trauma. and (4) stopped contact lens wear for ≥3 weeks for rigid gas permeable, and ≥ 1 week for soft contact lenses.

We included 272 NEs, 413 KC eyes and 72 PKC eyes from the Ming Vision Clinic (Clinic 1). We included 100 NEs, 87 KC eyes and 15 PKC eyes from the Fenglian JiaYue LeGe Clinic (Clinic 2). We included 72 NEs, 51 KC eyes and 14 PKC eyes from the Peking Union Medical College Hospital (Clinic 3). The patient data obtained from the clinics were used for the training model where input into a private model and not a public model.

### Datasets and pre-processing

Patients were divided into three datasets. The training set consisted of three groups. The NE group comprised 35 eyes from Clinic 1, 25 eyes from clinic 2, and 4 eyes from clinic 3. The KC group comprised 25 eyes from Clinic 2. The PKC group comprised 30 eyes from Clinic 1, five eyes from Clinic 2, and four eyes from clinic 3. The validation set comprised of three groups. The NE group comprised 15 eyes from Clinic 1, 15 from Clinic 2, and 4 from Clinic 3. The KC group comprised 15 eyes from Clinic 2. The PKC group comprised 10 eyes from Clinic 1, 5 eyes from Clinic 2, and 4 eyes from Clinic 3. The independent test group consisted of three groups. The NE group comprised 222 eyes from Clinic 1, 60 from Clinic 2, and 64 from Clinic 3. The KC group comprised 413 eyes from Clinic 1, 47 eyes from Clinic 2, and 51 eyes from Clinic 3. The PKC group comprised 32 eyes from Clinic 1, 5 eyes from Clinic 2, and 6 eyes from Clinic 3.

### Pre-processing of input images

Five standard topographical refractive maps (maps showing central corneal thickness, anterior (posterior) surface elevation, and anterior (posterior) surface curvature) were selected as the original data for our study. These maps were obtained from the Pentacam HR system and exported with a diameter of 8 mm. Each original image was then cropped to 540 × 540 × 3 pixels. We filtered out textual information and retained only color-gradient data from the exported images. The cropped images were then “stitched together” into a single row with dimensions of 2,700 × 540 × 3 pixels, following the order of maps of central corneal thickness, anterior (posterior) surface elevation, and anterior (posterior) surface curvature. This concatenation of five medical images allowed for joint learning, enabling the model to analyze related (but distinct) images simultaneously in one pass (11). This approach facilitated the identification of common features, alignments, correlations, and invariance across datasets.

To optimize training efficiency and minimize graphics processing unit (GPU) memory demands, we resized the input images to dimensions of 1,120 × 224 × 3 pixels. This resizing was performed to reduce the computational cost per iteration, to enable larger batch sizes, and to prevent out-of-memory errors. However, excessive downsizing can lead to performance degradation by eliminating discriminative details. Therefore, the chosen input dimensions were carefully selected to strike a balance between training speed and model accuracy.

In contrast to a conventional binary classification task that distinguishes between KC and NEs, we combined KC and PKC into one category to determine whether the model could identify the hidden distinctive features in PKC. To explain the possible impacts of interclass mixture and class imbalance in a multicenter study, we projected and analyzed corneal topographical images onto the feature space using t-distributed stochastic neighbor embedding (t-SNE), which helped design a strategy for the selection of training datasets that improved the detection accuracy of PKC as a form of KC against NE. We trained and optimized two models using the efficientnet-b0 architecture and the Train, Adapt, Optimize (TAO) Toolkit (NVIDIA, Santa Clara, CA, United States). The first model (referred to as “Model 1”) was trained using data from two centers. The second model (“Model 2”) used Model 1 as a pre-trained model and incorporated a small amount of additional data during training to enhance classification accuracy.

**Figure 1:**
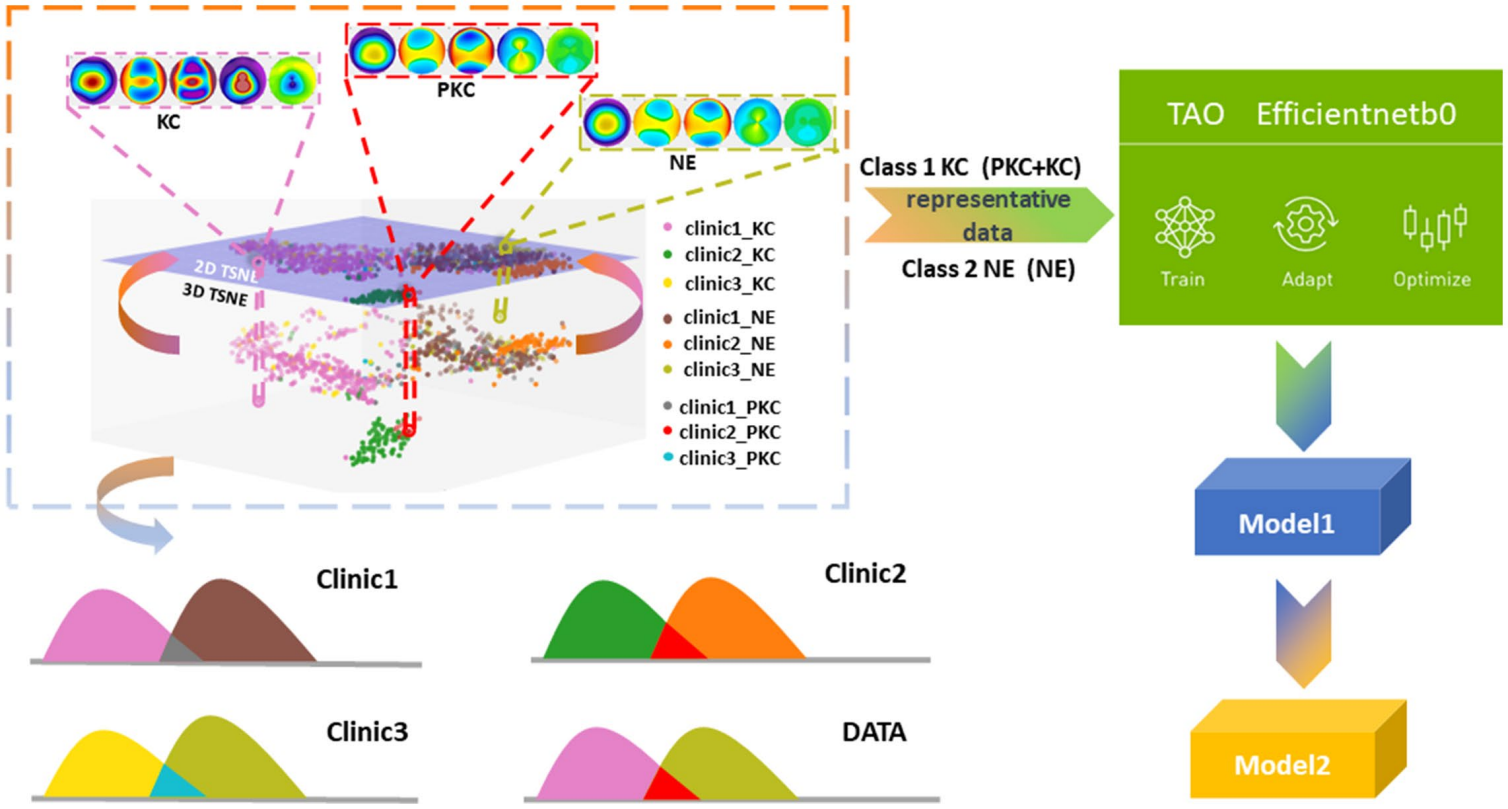
Illustrative diagram of domain differences due to multicenter data acquisitions.

In the present study, t-SNE was employed to analyze clinical data collected from multiple centers (12). The objective of this study was to project the spatial distributions of high-dimensional features of topographical images onto a two-dimensional scatter plot. The ground truths of the three class labels (KC, PKC, and NEs) were determined by two expert annotator. The results showed that the KC group (including the PKC group) was distinguishable from the NE group. Notably, the scattered KC points within the normal cluster were patients with PKC who shared visual similarity with NEs. Furthermore, the distributions of the corresponding classes between each center were scattered as separate clusters, indicating a domain shift (i.e., a mismatch caused by differences in the picture quality between images obtained from different tasks) (Figure 1). In our study, this mismatch arose from variations in export settings across multiple clinical centers. Consequently, the model training process could be confounded by the interclass mixture and the domain shift resulting from the acquisition of multicenter data, thereby posing challenges to the classification task.

### ML model

Our task involved distinguishing between two classes (PKC and KC) combined as one from the NE group using a small balanced dataset. Hence, we chose the TAO Toolkit (NVIDIA) (13) to train and optimize our model for the classification of KC/NEs. The TAO Toolkit is a low-code solution that allows non-AI professionals to expedite their processes for the training and optimization of a model. For this purpose, we selected efficientnet-b0 from TAO (14). This model employs a compound coefficient to scale network dimensions (e.g., depth, width, and resolution) uniformly. This balanced scaling approach results in improved performance compared with independent dimension scaling because it enables consistent growth of a network while maintaining a reasonable complexity of the model (15). Efficient net architectures achieve competitive accuracy with fewer parameters, owing to their sample efficiency resulting from balanced scaling (14). Therefore, the efficientnet-b0 model was well suited for our challenging binary classification task, which involved distinguishing between NEs and KC eyes, with limited subtle and readily confused cases.

### Training sets

During the training phase, we conducted a two-step process to train the two models based on the efficientnet-b0 model. Model 1 was a binary classification model trained on data from Clinics 1 and 2. The dataset used for training consisted of 40 PKC samples from Clinic 1, 40 KC samples, 10 PKC samples from Clinic 2, 50 NE samples from Clinic 1, and 40 NE samples from Clinic 2. To ensure representative samples, we employed a data selection strategy that curated balanced subsets of the KC, PKC, and NE subgroups from the collected datasets. The aim was to optimize the robustness of the efficientnet-b0 model. Model 2 was a binary classification model trained on the data from all three clinics. To enhance the performance of Model 2, we added 8 PKCs and 8 NEs from Clinic 3 to the training dataset for Model 1. Model 1 was used as a pre-training model, following the concept of transfer learning.

### Test sets

We employed two distinct test sets to evaluate the performance of the proposed two-step training model. The initial test set, referred to as “dataset 1,” comprised data obtained from two medical centers. Clinical 1 included 222 NEs, 413 KCs, and 32 PKCs. Clinic 2 included 60 NEs, 47 KCs, and 5 PKCs. The second test set, Dataset 2, encompassed data from three centers. It comprises data from Clinic 1 and Clinic 2 that were already present in Dataset 1, as well as 64 NEs, 51 KCs, and 6 PKCs from Clinic 3.

Given the limited availability of labeled data, we employed transfer learning to minimize the need for additional data when extending the model to adapt to data from new clinics (16). By providing a small sample from a new clinic, the pre-trained model can adapt quickly and maintain robust performance if deployed on larger, unseen datasets. As we repeated this process for additional clinics, the model progressively improved its versatility and robustness, despite the scarcity of data.

### The Belin-Ambrósio enhanced ectasia display total deviation

The Belin-Ambrósio enhanced ectasia display total deviation (BAD-D) score was used as a diagnostic index by the Pentacam HR system. According to the Pentacam HR system, normal eyes are distinguished from the KC or KC suspects when the total D < 1.6, the standard have been used in various studies related to keratoconus or corneal ectasia (17–20). Ambrósio et al (17) used the index (total D > 1.6) as the risk factors for post-LASIK ectasia development. Herber et al. (18), Wang et al. (19) and Koc et al. (20) used the index (total D < 1.6) to help distinguish normal eyes from keratoconic eyes. In our study, cases from test sets with the total D < 1.6 were labelled “normal” as the diagnosis results of BAD-D. Finally, we compared the classification results of test sets of Model 2 with the BAD-D by the area under the receiver operating characteristic (ROC) curve (AUC). The sensitivity and specificity were also calculated on the basis of the test results of the Model 2 and the BAD-D to compare the two diagnostic tools.

### Reading protocol and study groups

Two expert ophthalmologists were invited to make standard annotations for the images. Five junior ophthalmologists were trained and read the images for assessment in the study. The junior ophthalmologists included in the research were ophthalmologists who have accepted training of ophthalmology <5 years. Five junior ophthalmologists were selected from the Clinic 1. Before the formal annotation, they were trained on label principles. The diagnostic criteria of the disease and their clinical characteristics were not included in the training to maintain the real diagnostic capacity of junior ophthalmologists in clinical practice. After the training phase, each of the junior ophthalmologists was assigned to annotate portions of the datasets independently as the control group; specifically, each ophthalmologist annotated images obtained from the same test set as the ML model in Clinic 1. Then, they annotated the same groups of images, attached with labels previously annotated by the ML model, forming the test group. At last, all the junior ophthalmologists were request to sign up a questionnaire to evaluate the assistance of the ML model.

### Statistical analyses

Data distribution was evaluated using the Kolmogorov–Smirnov goodness-of-fit test. Comparisons between groups were performed using the nonparametric Kruskal–Wallis test, followed by the *post-hoc* Dunn’s test to compare each pair of groups. We also computed confusion matrices and accuracy to objectively compare the performance and quality of learning. DeLong’s test was performed to pairwise compare different AUCs, and the binomial exact test was used to calculate 95% confidence intervals. Statistical analyses were performed using SPSS version 20 (IBM, Armonk, NY, United States).

## Results

### Study population

The mean age was 27.5 ± 7.6 years in the KC group and 28.1 ± 6.9 years in the NE group (*p* < 0.05). The mean K1 was 46.2 ± 6.0 in the KC group and 42.9 ± 1.4 in the NE group (*p* < 0.05). The mean K2 was 49.4 ± 7.0 in the KC group and 44.3 ± 1.6 in the NE group (*p* < 0.05). The mean value for astigmatism was 3.1 ± 2.5 in the KC group and 1.4 ± 0.8 in the NE group (*p* < 0.05). The mean Pachy min value was 463.8 ± 53.9 in the KC group and 534.8 ± 29.7 in the NE group (*p* < 0.05). The mean I-S (inferior–superior) was 4.4 ± 3.9 in the KC group and 0.3 ± 0.7 in the NE group (*p* < 0.05). The parameters for the different sets are listed in Table 1. In cases of severe KC, the K value and Pachy min values were unusual because the cornea was extremely distorted (Supplementary Figures S1–S3).

**Table 1:** Descriptive statistics of train and test sets.

	NE train set				PKC train set				KC train set			
	Mean	SD	Min	Max	Mean	SD	Min	Max	Mean	SD	Min	Max
Age	26.8980	6.94082	10.00	38.00	26.1724	6.45942	13.00	42.00	27.3750	5.64182	18.00	42.00
K1	43.0316	1.28589	40.40	45.70	43.2828	2.31381	38.60	56.80	47.1725	5.83086	38.20	70.40
K2	44.3592	1.46919	40.80	48.10	44.7103	2.44868	39.60	57.80	49.8425	6.85116	38.80	76.90
Astig F	1.3255	0.63996	0.10	2.70	1.4276	0.79423	0.20	4.70	2.6675	1.89323	0.00	6.50
Num. Ecc. F	0.5621	0.13168	0.04	0.90	0.5971	0.18599	−0.07	1.38	0.7830	0.49776	−0.82	1.52
Asph. Q F	−0.3528	0.14856	−0.82	−0.02	−0.4041	0.25318	−1.89	0.10	−0.8353	0.66461	−2.33	0.69
Num. Ecc. B	0.4931	0.15344	0.04	0.77	0.5790	0.17064	0.19	1.41	0.8650	0.38945	−0.07	1.45
Asph. Q B	−0.3323	0.13314	−0.68	−0.05	−0.4074	0.25162	−2.00	−0.15	−0.9435	0.59889	−2.10	−0.01
Pachy Apex	542.3673	29.92444	477.00	615.00	506.2241	32.90925	409.00	602.00	460.7500	49.05870	328.00	561.00
Pachy Min	539.3367	29.73108	475.00	614.00	502.2414	32.78977	408.00	598.00	454.7500	48.32622	325.00	554.00
ISV	18.4082	5.44672	7.00	37.00	24.0172	15.68941	11.00	131.00	88.9500	48.60619	19.00	195.00
IHD	0.0122	0.00628	0.00	0.03	0.0210	0.01868	0.00	0.14	0.1250	0.08153	0.02	0.33
KMax	44.9480	1.50937	41.10	48.40	45.8707	4.12230	40.00	72.80	57.3425	9.99230	42.40	83.20
IS-value	0.3980	0.66631	−0.98	1.82	0.8638	0.97522	−1.44	4.89	5.5620	3.96846	0.37	19.62

	NE test set				PKC test set				KC test set			
	Mean	SD	Min	Max	Mean	SD	Min	Max	Mean	SD	Min	Max
Age	28.5029	6.87581	9.00	46.00	27.0233	6.93675	13.00	46.00	27.7319	7.94938	9.00	63.00
K1	42.9390	1.47774	39.10	47.20	42.4605	1.24750	39.30	45.10	46.8722	6.37117	24.70	75.60
K2	44.4000	1.62393	40.00	49.50	44.1651	1.31219	39.80	48.10	50.4299	7.26668	36.70	84.60
Astig F	1.4558	0.84913	0.00	5.30	1.7047	0.98970	0.20	5.90	3.5579	2.65677	0.00	19.00
Num. Ecc. F	0.5526	0.11157	0.18	0.92	0.5644	0.21396	−0.51	0.85	0.8020	0.47790	−1.08	1.71
Asph. Q F	−0.3353	0.11875	−0.87	−0.07	−0.3707	0.18409	−0.80	0.32	−0.8682	0.69719	−4.47	2.25
Num. Ecc. B	0.5201	0.14955	0.04	0.91	0.5200	0.17829	0.02	0.86	0.8966	0.37724	−0.76	4.35
Asph. Q B	−0.3595	0.14315	−0.90	−0.06	−0.3498	0.17185	−0.79	−0.03	−1.0209	1.58432	−34.34	1.37
Pachy Apex	537.7457	27.91497	479.00	613.00	514.7209	26.32881	426.00	559.00	466.0078	54.04653	177.00	718.00
Pachy Min	534.4364	27.77274	471.00	611.00	509.1628	26.51408	423.00	556.00	456.2896	54.22577	64.00	654.00
ISV	18.7890	6.09433	6.00	45.00	25.0465	10.01892	13.00	63.00	86.4070	48.01600	14.00	352.00
IHD	0.0116	0.00633	0.00	0.04	0.0230	0.01809	0.01	0.08	0.1177	0.07753	0.00	0.45
KMax	44.9780	1.64484	40.70	50.20	45.4326	1.76578	41.40	52.10	57.0724	10.77521	41.50	100.80
IS-value	0.3267	0.67708	−1.72	2.41	1.1947	1.07782	−0.57	4.39	5.0416	3.90189	−8.97	23.29

### ML model characteristics

The accuracy of the ML model between the eyes of patients with KC and NEs in all three clinics (99% accuracy, area under the receiver operating characteristic (ROC) curve AUC of 1.00, 99% sensitivity, 99% specificity). The accuracy of the ML model between eyes with PKC and NEs in all three clinics (98% accuracy, AUC of 0.96, 98% sensitivity, 98% specificity). The sensitivity and specificity of ML model is higher than that of the previous studies (21–30) shown in Table 2.

**Table 2:** The sensitivity and specificity of the previous PKC studies.

	Bühren et al. (21)	Chan et al. (22)	Kovács et al. (23)	Saad et al. (24)	Hidalgo et al. (25)
Sensitivity	78.10%	70.80%	90%	63%	79.10%
Specificity	83.30%	98.10%	90%	82%	97.90%
	Hidalgo et al. (26)	Xu et al. (27)	Ambrósio et al. (28)	Steinberg et al. (29)	Shi et al. (30)
Sensitivity	61%	83.70%	90.40%	63%	98.50%
Specificity	75%	84.50%	96%	83%	94.70%

**Figure 2:**
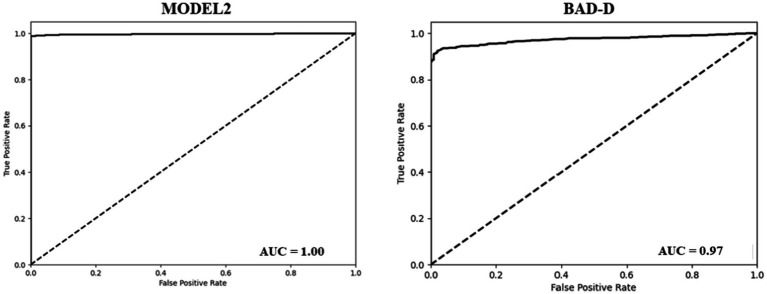
ROC curve of the model 2 (Left) and ROC curve of the BAD-D (Right) for KCN diagnosis.

The BAD-D is a widely used reference for assessing the KC (31). It calculates a total deviation value (known as a global “D”) by analyzing specific parameters and comparing them with a normative database. Our dataset was classified into NE and KC groups based on the BAD-D criteria, with a cut-off threshold of 1.6 (26). The BAD-D achieved an accuracy of 0.86, a sensitivity of 0.97, and a specificity of 0.69. The AUC of the BAD-D was 0.97, which was lower than that of model 2 (Figure 2). In comparison, Model 2 demonstrated significantly improved classification metrics compared to BAD-D. It achieved a 13% higher accuracy, 2% higher sensitivity, and a substantial 30% improvement in specificity. The higher specificity of model 2 indicated a lower false-positive rate. These results highlight the superior discriminative capability of Model 2 relative to the BAD-D, particularly for challenging borderline cases.

**Figure 3:**
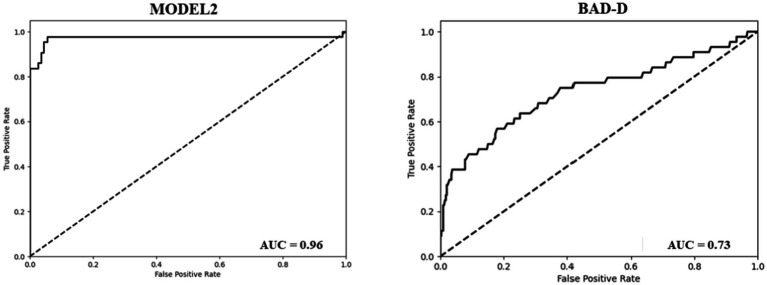
ROC curve of the model 2 (Left). ROC curve of the BAD-D for the classification of PKC and NE (Right).

To assess the effectiveness of our model in identifying cases of PKC accurately, we further divided the results into two classes (PKC and KC) and demonstrated the classification results of PKC and NE using Models 2 and BAD-D. Model 2 accurately categorized 42 of the 43 PKCs in Dataset 2. In contrast, BAD-D correctly identified only 29 of 43 PKC cases, indicating its limited ability to discriminate early-stage disease. In addition, Model 2 achieved high accuracy in classifying the NE cases. In comparison, the BAD-D misclassified 106 NE cases. The AUC of the BAD-D was 0.73, which was significantly lower than that of Model 2 (Figure 3). Model 2 demonstrated superior discriminative ability over the BAD-D in distinguishing challenging borderline PKC and NE cases.

### *Post-hoc* analysis

**Figure 4:**
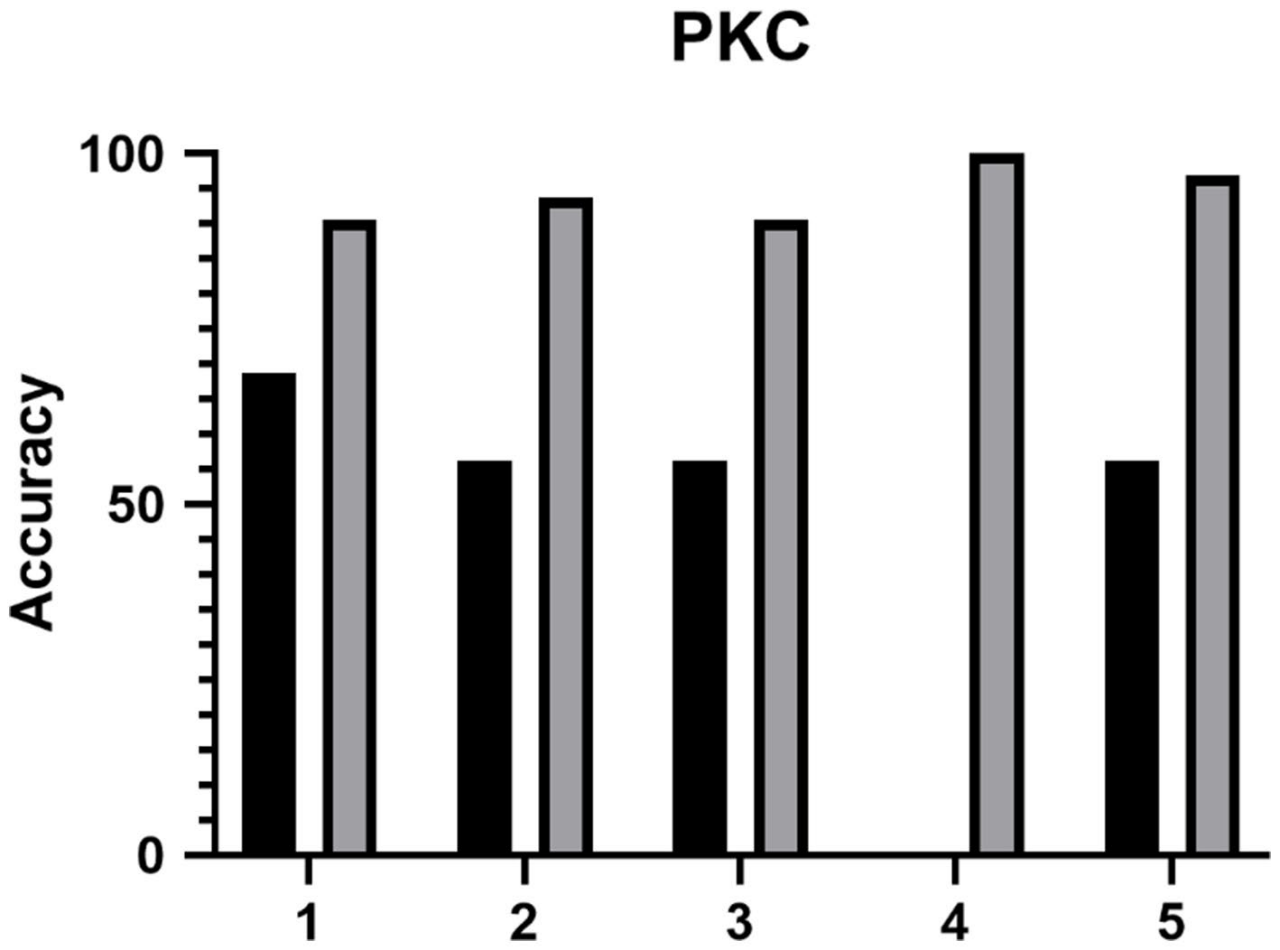
The diagnostic accuracy of the five junior ophthalmologists (1–5) of PKC before and after the training of ML model.

The accuracy of the ML model between the eyes of patients with KC and NEs in clinic 1 was 98.74 (97.86% sensitivity, 100% specificity). The accuracy of the ML model between the eyes of patients with PKC and NEs in clinic 1 was 96.85 (72.72% sensitivity, 100% specificity). The diagnostic accuracy for junior ophthalmologists to detect PKC was 47.5% (95%CI, 0.5–71.6%), 100% (95%CI, 100–100%) and 94.4% (95%CI, 14.7–94.7%) in the control group, ML model group and test group. With the ML model training, the diagnostic accuracy of junior ophthalmologists improved with statistical significance (*p* < 0.05) as shown in Figure 4. According to the questionnaire, the average score (total 5) of the questionnaire was: 1. How do you think AI models help you understand the complexity of image judgment in PKC? (3.8) 2. How do you think AI models help you understand the complexity of image judgment in severe keratoconus? (4) 3. How comprehensive do you think the AI model has been in learning your keratoconus diagnosis? (4) 4. How convenient do you think the AI model is for you to learn the diagnosis of keratoconus? (4.4) 5. How do you think AI models help you learn to judge keratoconus as a whole? (3.6).

### Typical PKC cases

**Figure 5:**
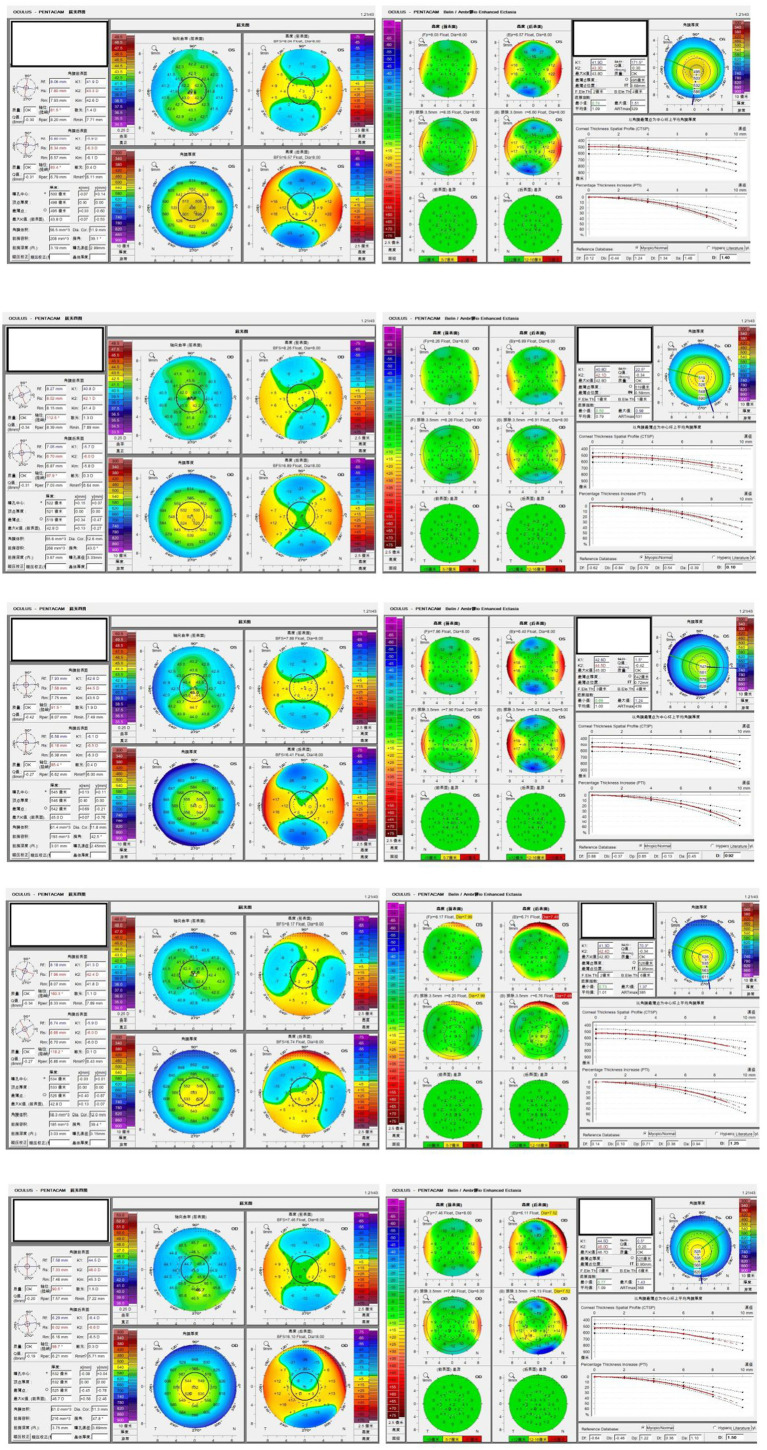
Pentacam images of typical PKC patients 1–5.

The Pentacam HR images of five typical patients with PKC are shown in Figure 5. These cases were difficult to detect as PKC, and they were misdiagnosed as NEs by BAD-D. However, they were correctly identified using ML model.

## Discussion

Detection of PKC is a difficult clinical problem. Researchers have developed various models based on ML to aid the diagnosis of PKC, but the results have been unsatisfactory (21–27, 29).

In some studies, sensitivity and specificity were relatively high. However, this study had several limitations. In a study by Ambrósio et al. (28), although the test results were high, they used Pentacam combined with Corvis^®^, which is not commonly used worldwide, thereby limiting the application of the model. The study by Shi et al. (30), they had the same limitations. They used a prototype of ultra-high-resolution optical coherence tomography, which has not been used in clinical practice, thereby endowing their model with limited practical value.

The model was trained by the Efficientnet-b0 net, which architectures have been confirmed to achieve competitive accuracy with fewer parameters, owing to their sample efficiency resulting from balanced scaling. From the review of the previous study, we can see that the sensitivity of the machine learning model to detect PKC is relatively higher. Also, rather than using multiple diagnosis devices, the model only utilized pentacam HR system to screen PKC, making the model with more practicability. We aimed to make the model more applicable because the Pentacam HR is commonly used to detect PKC by refractive surgeons globally.

By combining KC and PKC cases from three clinics, we included 551 KC eyes and 101 PKC eyes. Hence, this is one of the largest ML studies on KC and PKC. Depending only on Pentacam HR images, the model of our study could reach 99% accuracy, 99% sensitivity, 99% specificity, and an AUC of 1.00 to distinguish KC and NEs, much higher than those for BAD-D (86% accuracy, 97% sensitivity, 69% specificity, AUC of 0.97). The advantage of our model is the more prominent distinction between PKC and NEs. In this regard, the model could reach 98% accuracy, 98% sensitivity, and 98% specificity, with an AUC of 0.96, which was remarkably higher than those achieved using the BAD-D (69% accuracy, 67% sensitivity, 69% specificity, AUC of 0.73), making it one of the best ML models to distinguish PKC from NEs.

In contrast to a conventional training strategy (32, 33), we used a pre-trained model optimized using the TAO Toolkit. Our model was trained on a small (yet evenly balanced) training set to achieve the highest accuracy for the classification of PKC and NEs. The model was trained with only 25 KCs, 64 NEs, and 39 PKCs, modified in the validation set of 15 KCs, 34 NEs, and 19 PKCs, but obtained 99% accuracy and an AUC of 1.00 in a much larger test set (511 KCs, 346 NEs, 43 PKCs). The traditional training pattern usually uses 80% of the cases to train the model and tests only 20% of the cases. The pre-trained model that we describe can test much larger datasets by only training very small data, making the model more widely applicable in clinical practice.

In the research, with the help of the proposed ML model, the diagnostic accuracy of junior ophthalmologists to detect PKC improved significantly, indicating that the model may be potential tool for diagnosing PKC in clinic. Compared with the traditional training model, the model can achieve high accuracy with minimum training data. In addition, the model relies only on Pentacam HR images, making it very valuable for practical applications.

The machine learning model can provide high-level detection of PKC, making it potential assistant of refractive surgeons to screen qualified candidates for corneal refractive surgery. The model is of favourable practicability, since it can provide screening results only by using single diagnosis device pentacam HR system. However, more independent data from different clinics should be test to confirm the stability of the machine learning model.

## Data Availability

The raw data supporting the conclusions of this article will be made available by the authors, without undue reservation.
