# Supplementary figures and images for: The development of a machine learning model to train junior ophthalmologists in diagnosing the pre-clinical keratoconus
Source: Front Med (Lausanne). 2024 Sep 18;11:1458356. doi: 10.3389/fmed.2024.1458356 (PMC11445185; doi:10.3389/fmed.2024.1458356)

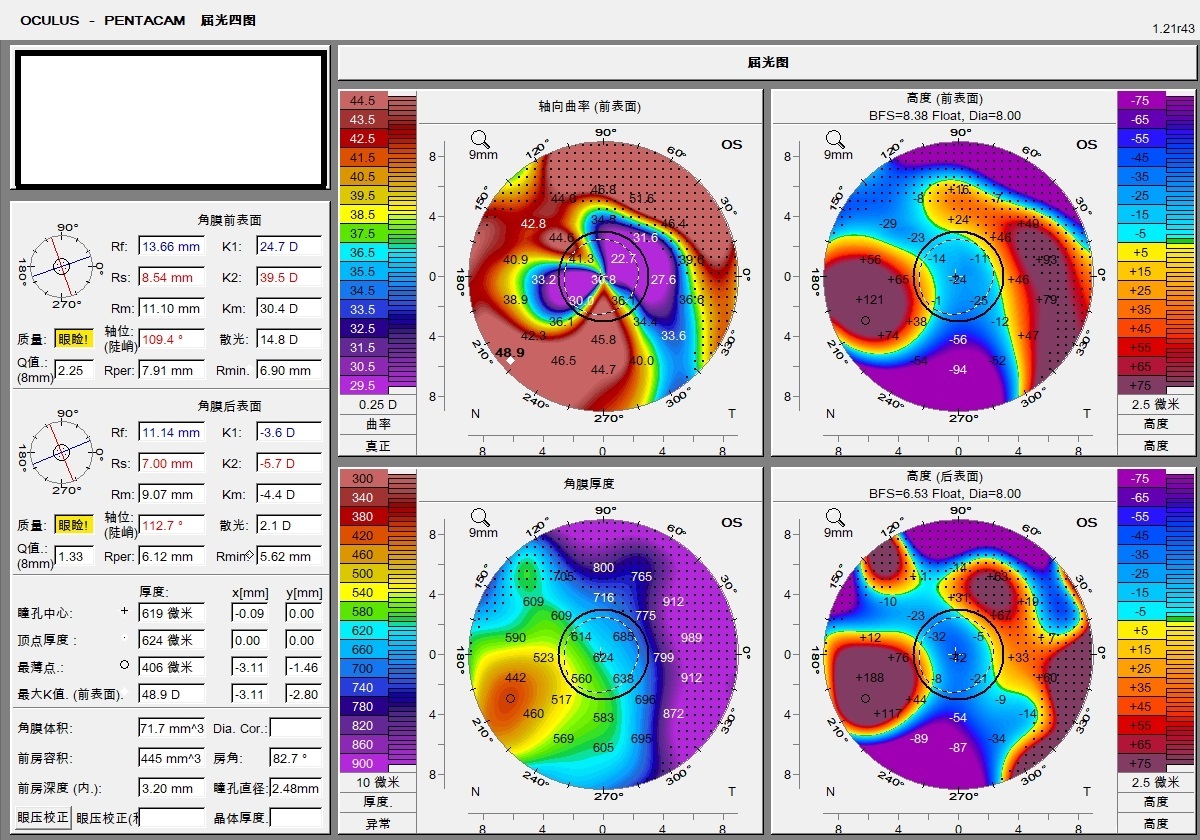

Supplement: Supplementary file 1 [file Image_1.JPEG]

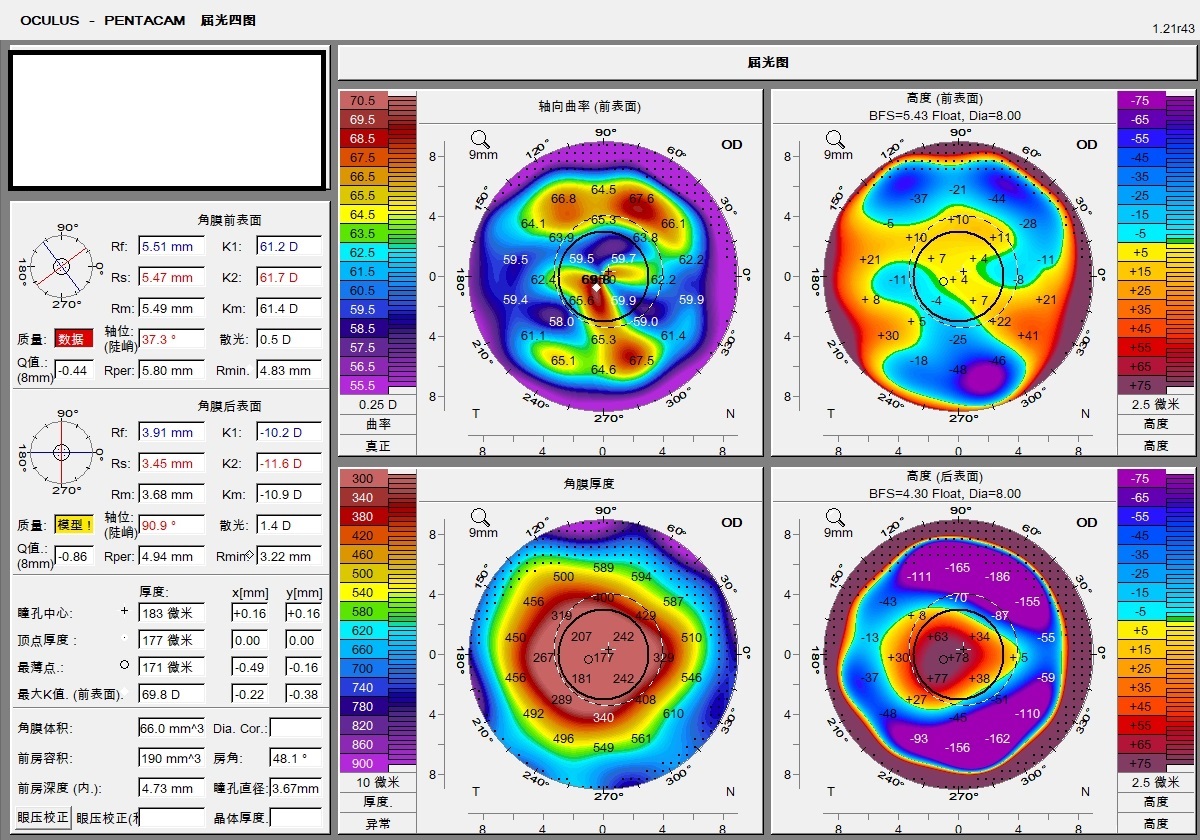

Supplement: Supplementary file 2 [file Image_2.JPEG]

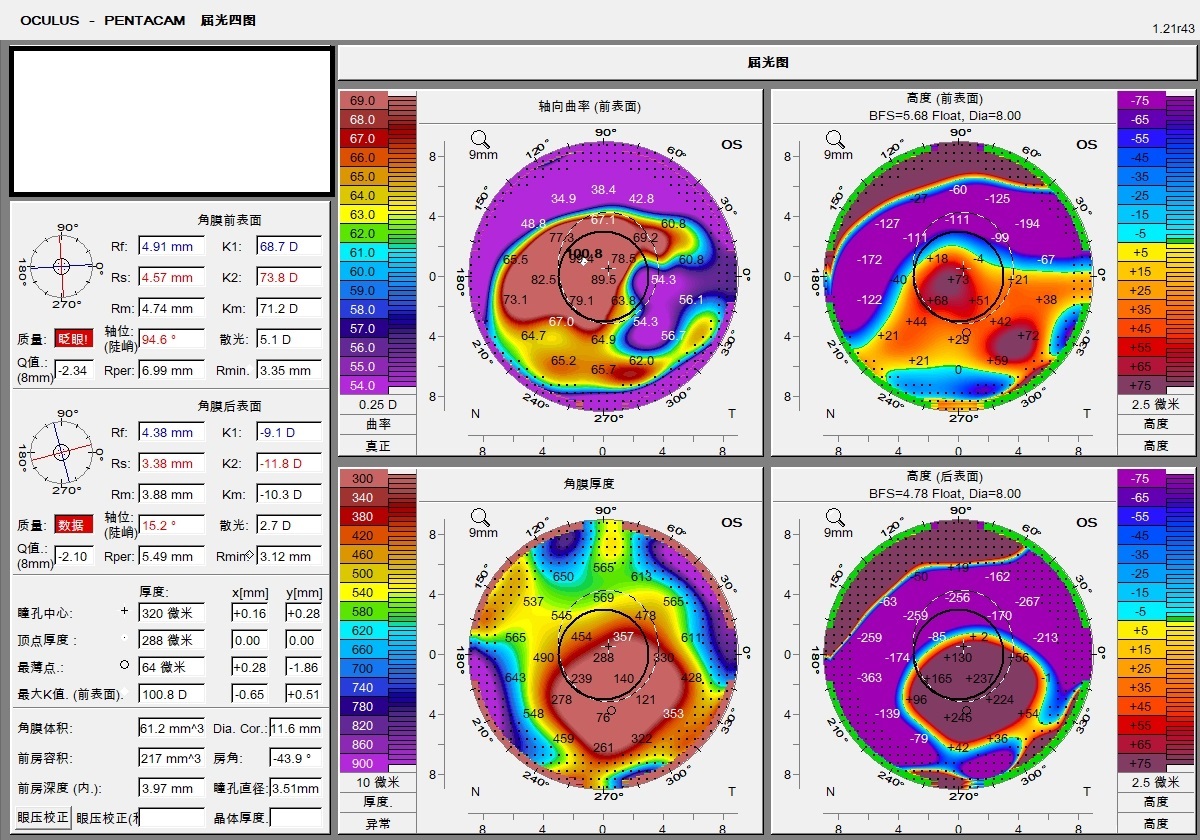

Supplement: Supplementary file 3 [file Image_3.JPEG]
